# Temporary Transvenous Pacemaker for the Treatment of Diphtheria Myocarditis and Progressive Conduction Block: A Case Report

**DOI:** 10.7759/cureus.65508

**Published:** 2024-07-27

**Authors:** Khadijah Maghrabi

**Affiliations:** 1 Department of Pediatrics, King Abdulaziz University, Jeddah, SAU

**Keywords:** pediatrics, myocarditis, conduction abnormalities, atrioventricular block, diphtheria

## Abstract

A case of severe respiratory diphtheria complicated by myocarditis is reported. Diphtheria myocarditis manifested as cardiogenic shock and progressive conduction disturbance. Temporary transvenous ventricular pacing was used to manage this complication successfully. However, this patient died from cardiogenic shock. This case highlights that sporadic cases of diphtheria still occur despite high vaccination rates in Saudi Arabia. Conduction abnormalities in diphtheria myocarditis carry a prognostic marker for the severity of cardiac injury.

## Introduction

Diphtheria is an acute fatal bacterial disease caused by toxigenic *Corynebacterium diphtheriae*. Respiratory diphtheria manifests as sore throat, malaise, and pharyngeal erythema, which can progress to the formation of a white-grayish exudate. Absorption and dissemination of diphtheria toxin leads to systemic manifestations: myocarditis, neuropathy, acute kidney injury (AKI), and multiorgan failure [1]. It primarily affects children with particular affinity to unimmunized and immunocompromised individuals [2]. Cardiac manifestation, in the form of myocarditis, is the most serious involvement of diphtheria and is the most common cause of mortality in infected patients [3,4]. Diphtheritic myocarditis manifests as heart failure, cardiogenic shock, atrial or ventricular arrhythmias, or more rarely conduction system abnormalities [5,6]. We present a case of diphtheric myocarditis in a nine-year-old female, complicated with progressive atrioventricular (AV) block requiring rescue transvenous pacing.

## Case presentation

A nine-year-old female presented to the emergency room with a fever and sore throat for two days. She was previously in good health, but she had not been immunized due to parental refusal. In the emergency room, she was febrile and in respiratory distress. Her vital signs are as follows: heart rate, 128/minute; blood pressure, 97/61; respiratory rate, 30/minute; temperature, 39°C; and oxygen saturation, 98% on room air. She was sitting upright with her neck extended forward and drooling. She was noted to have tender neck swelling with palpable painful cervical lymph nodes. Throat examination showed enlarged tonsils with an extensive grayish-white pseudomembrane overlying the tonsils and pharynx. In view of a compromised airway, the patient was transferred to the operating room where endotracheal intubation was done. Upon visualization of the airway, there was extensive pharyngeal exudate but no significant airway narrowing, and intubation was done easily without complications. Upon arrival to the intensive care unit, the patient had developed metabolic acidosis with cool extremities, although systolic blood pressure was still adequate. She was resuscitated with fluid boluses and broad-spectrum antibiotics (meropenem and vancomycin). Pharyngeal swab cultures were negative, but the sample was not grown on specific media for *Corynebacterium diphtheriae* due to its lack of availability. However, given the high clinical suspicion of the clinical findings of diphtheria, diphtheria anti-toxin was administered on the fourth day. This delay was due to a lack of immediate availability. Over the next couple of days, she continued to have worsening hemodynamics, and epinephrine and norepinephrine were started for progressive hypotension. Serial echocardiogram showed progressively worsening systolic function (initial echocardiogram showed an ejection fraction of 50%, which progressed to 25% within 48 hours). She also had elevated troponin values. She also had a raised white blood count and C-reactive protein, and gradually worsening urea and creatinine (Table 1).

**Table 1:** Laboratory results on admission and on the day of death

Test		Result on admission	Result on day 7 of admission	Reference range
Complete blood count	White blood cells	27.6 K/uL	47.4 K/uL	4.5-13.5 K/uL
	Automated neutrophil count	21.9 K/uL	36.6 K/uL	1-8.5 K/uL
	Automated lymphocyte count	2.4 K/uL	4.47 K/uL	1.5-6.8 K/uL
	Red blood cells	4.89 M/uL	3.14 M/uL	4-5.40 M/uL
	Platelets	298 K/uL	292 K/uL	150-450 K/uL
	Hemoglobin	12.0 g/dL	7.9 g/dL	12-15 g/dL
	Hematocrit	36.1%	24.5%	35%-49%
	Mean cell volume	73.8 fL	78 fL	80-96 fL
	Mean cell hemoglobin	24.5 pg	24.2 pg	32-36 pg
Coagulation profile	Prothrombin time	14 seconds	58.3 seconds	10-13 seconds
	Activated partial thrombin time	36.2 seconds	57 seconds	25.1-36.5 seconds
	D-dimer	5.45 mg/L	>20 mg/L	0-0.5 mg/L
	International normalized ratio	1.05	4.61	0.85-1.3
Renal function and electrolytes	Potassium	4.3 mmol/L	5.8 mmol/L	3.5-5.1 mmol/L
	Sodium	138 mmol/L	139 mmol/L	136-145 mmol/L
	Chloride	110 mmol/L	105 mmol/L	190-107 mmol/L
	Urea	6.6 mmol/L	20.4 mmol/L	2.5-6.4 mmol/L
	Creatinine	50 µmol/L	358 µmol/L	53-115 µmol/L
Liver function test	Aspartate aminotransferase	50 U/L	-	15-37 U/L
	Alanine aminotransferase	16 U/L	-	12-78 U/L
	Gamma-glutamyl transferase	10 U/L	-	5-85 U/L
	Total bilirubin	6 U/L	-	0-17 U/L
	Total protein	69 g/L	-	64-82 g/L
	Albumin	42 g/L	30 g/L	40-47 g/L
	C-reactive protein	165 mg/L	47.7 mg/L	0-3 mg/L
	Lactic acid	0.6 mmol/L	11 mmol/L	0.5-2.2 mmol/L
	Lactate dehydrogenase	-	>4,500 U/L	120-246 U/L
	Troponin I	16.74 ug/L	47.51 ug/L	0.02-0.04 ug/L

**Figure 1:**
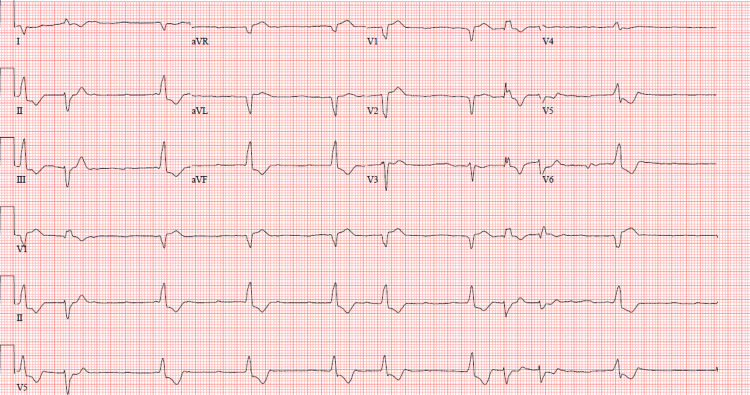
12-lead ECG showing complete AV block with wide QRS escape idioventricular rhythm and PVCs ECG: electrocardiogram, AV: atrioventricular, PVCs: premature ventricular contractions

**Figure 2:**
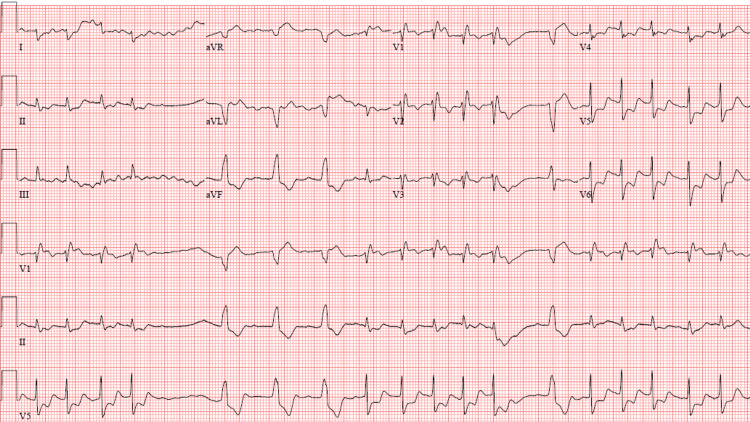
12-lead ECG showing runs of ventricular tachycardia on a background of complete AV block ECG: electrocardiogram, AV: atrioventricular

Continuous renal replacement therapy (CRRT) was started for acute kidney injury (AKI) and anuria on day 5. The first electrocardiogram (ECG) showed sinus rhythm with right bundle branch block; however, follow-up ECGs over the next few days showed progressive impaired conduction, starting with right bundle branch block and ending ultimately with complete atrioventricular block alternating with runs of ventricular tachycardia (Figure 1 and Figure 2). No electrolyte abnormalities were present at the time of these progressive ECG changes.

**Figure 3:**
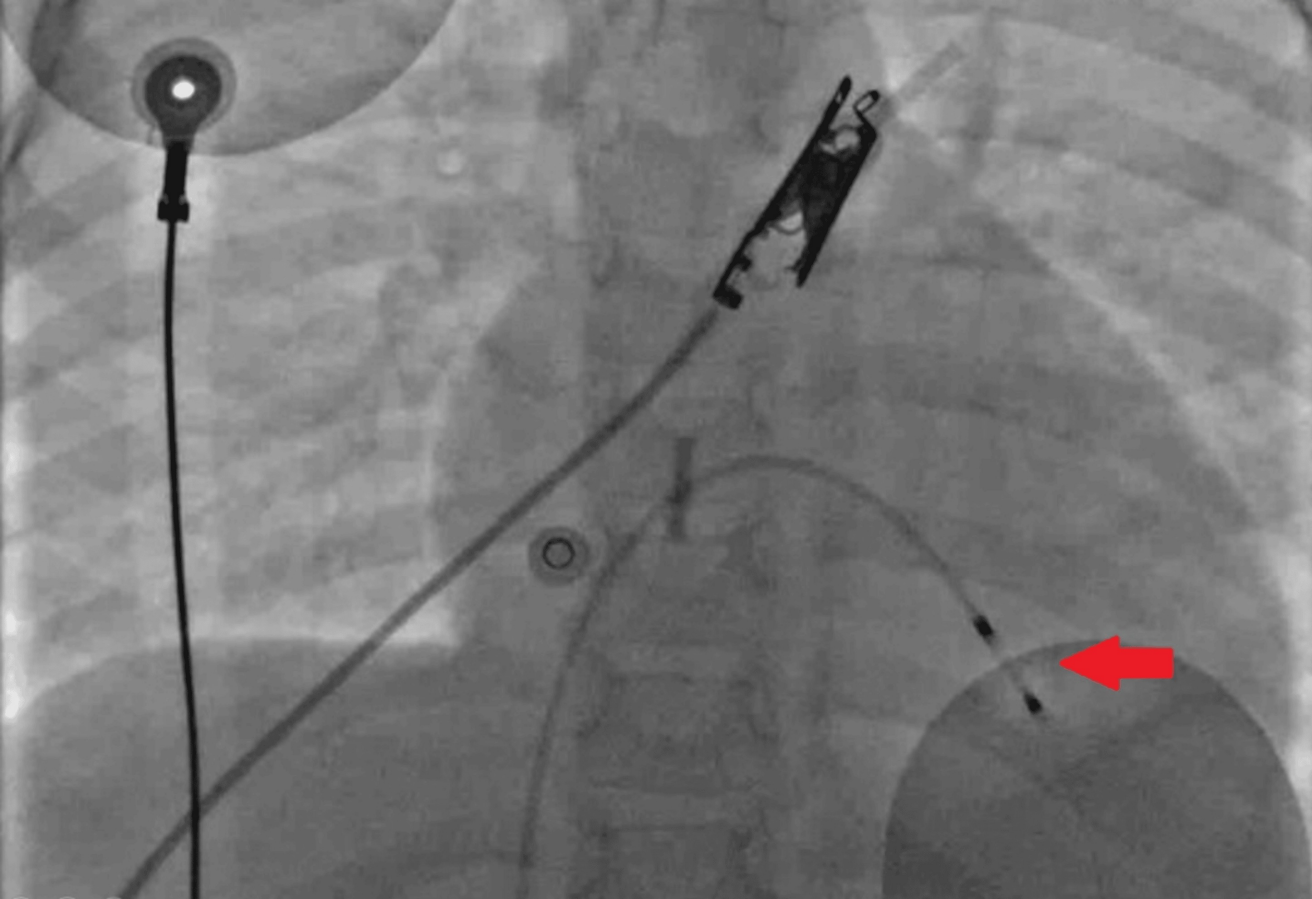
Temporary transvenous pacing catheter inserted through femoral vein access and positioned at the apex of the right ventricle (arrow)

She underwent transvenous pacemaker catheter insertion through the right femoral vein for temporary pacing (Figure 3). The pacing catheter was positioned at the apex of the right ventricle (RV) in a stable position. Capture threshold was 1.0 V at 1.0 msec. Temporary ventricular pacing was 120 paces/minute, with good capture.

Pulse methylprednisolone was started on the fifth day, but despite that, she continued to have worsening cardiogenic shock, with multiorgan dysfunction, AKI, and disseminated intravascular coagulation. Unfortunately, extracorporeal membrane oxygenation was not available, and the patient died 48 hours after pacing (seven days after admission).

## Discussion

Epidemiology

Diphtheria is a vaccine-preventable disease that primarily infects children with high morbidity and mortality rates. Although vaccination programs introduced in the 1940s and 1950s have succeeded in reducing the incidence of diphtheria, sporadic cases continue to occur in developing countries, particularly in non-vaccinated individuals [7]. The reemergence of diphtheria has also been noted in Eastern European countries, India, Africa, and the Middle East [8,9]. Despite high vaccination rates among children in Saudi Arabia [10], diphtheria cases continue to be reported in non-vaccinated populations [11].

Diphtheria diagnosis and cardiotoxicity

The clinical syndrome is attributed to the diphtheria toxin that affects multiple systems, including cardiac, airway, respiratory, renal, disseminated intravascular coagulation, and neurological [1]. A study conducted by Jayashree et al. [4] revealed that a paucity of immunization, hypotension during admission, and the existence of complicating factors such as respiratory obstruction, myocarditis, and renal insufficiency had a noteworthy detrimental impact on the disease prognosis. Cardiac manifestation in diphtheria occurs in 10%-20% of the cases but is frequently the most common cause of fatality [12]. Cardiac involvement occurs in the form of myocarditis in about 16% [13] up to 60% in some reports [4]. Other manifestations include ST-segment elevation, atrial and ventricular arrhythmias, and conduction abnormalities. Clinically significant conduction abnormalities including complete AV block occur in approximately 50% and are nearly always fatal [14,15].

Postmortem myocardial examination in these patients shows inflammatory exudate, loss of myofibrils with areas of extensive granular degeneration, and loss of cross striation [16]. The valves, coronaries, epicardium, and endocardium are typically preserved. The extent of changes is related to the cumulative exposure to diphtheria toxin. Diphtheria toxin causes deoxyribonucleic acid (DNA) fragmentation and inhibits eukaryotic elongation factor 2 (EEF2) activity, which contributes to a critical step in protein synthesis [6]. Kadyrov et al. [17] reported on 102 patients who died of diphtheria cardiotoxicity and showed dystrophic-necrotic changes in the cardiac conduction system between days 1 and 8, ending with myocardiosclerosis.

Conduction abnormalities in patients with diphtheria myocarditis are indicative of the severity of myocardial injury and are associated with a high fatality rate [15]. It is unknown whether rescue pacing improves survival in patients with diphtheria myocarditis and AV block. Matisonn et al. [18] reported successful pacing for a child with a complete AV block in South Africa. Dung et al. [19] reported a reduction in the mortality rate to 74% with the insertion of a temporary pacemaker for AV block. Other studies, such as a 10-year retrospective analysis of 46 patients with diphtheria myocarditis, suggested that cardiac pacing is not associated with improved survival in patients with complete AV block and bundle branch block [13]. This unfavorable response to pacing is likely related to the severe systolic dysfunction and significant myocardial injury that accompany conduction abnormalities [13]. Therefore, even with electrical pacing, the cardiac output remains severely inadequate, and patients die of cardiogenic shock.

Our case of highly suspected diphtheria demonstrates evidence of severe myocarditis and progressive conduction impairment. Due to the delayed availability of diphtheria anti-toxin, it was given on the fourth day of illness. Despite rescue ventricular pacing, she died of progressive cardiogenic shock. This case's dismal outcome encourages healthcare providers to earlier diphtheria anti-toxin administration to potentially improve outcomes for patients experiencing severe complications.

## Conclusions

Diphtheria myocarditis is an important and highly lethal complication of diphtheria that occurs in non-vaccinated children. Conduction system abnormalities are common in patients with diphtheria myocarditis and are markers of severe myocardial damage, with poor response to pacing. Prompt diagnosis and early administration of diphtheria anti-toxin and proper supportive care are currently the best available interventions.
